# Research Status of Molecular Dynamics Simulation of Metallic Ultrasonic Welding

**DOI:** 10.3390/mi16101185

**Published:** 2025-10-20

**Authors:** Yu Hu, Huan Li

**Affiliations:** 1 School of Locomotive and Vehicle Engineering, Guangzhou Railway Polytechnic, Guangzhou 511300, China; huyuscnu@163.com; 2 School of Mechanical Engineering, Yangtze University, Jingzhou 434023, China; 3 State Key Laboratory of Low Carbon Catalysis and Carbon Dioxide Utilization, Wuhan 430100, China

**Keywords:** ultrasonic welding, molecular dynamics simulation, process parameters, welding quality, sustainable development

## Abstract

This study provides a comprehensive review of ultrasonic welding research in molecular dynamics simulations, encompassing the latest advancements by scholars worldwide. Compared to traditional welding methods, ultrasonic welding offers advantages such as faster processing speed, higher mechanical strength, and environmentally friendly characteristics. However, its process parameters are subject to multiple influencing factors. Molecular dynamics simulations enable the detailed visualization of material interactions and structural changes at atomic/molecular levels during ultrasonic welding. These simulations not only predict how different process parameters affect weld quality but also facilitate the rapid identification of viable solutions, thereby reducing experimental iterations and lowering R&D costs. This review delves into the core theoretical issues pertaining to ultrasonic welding, providing robust support for practical applications. Additionally, specific optimization strategies are proposed to enhance welding performance and efficiency, promoting sustainable development in related industries. Future research could focus on exploring ultrasonic welding mechanisms under complex structures and multi-component systems.

## 1. Research Background and Significance

### 1.1. Research Background

With the rapid advancements in material bonding technologies, welding has emerged as a pivotal connection method, playing a vital role across multiple industries, including electronics, automotive, and aerospace. Ultrasonic welding technology has gained prominence as a research focus due to its efficiency, energy efficiency, and environmental benefits. However, the welding process involves complex physical and mechanical phenomena, particularly at the atomic and microscopic scales. Critical challenges such as material diffusion behavior, interface structure evolution, and factors affecting welding quality have become key constraints hindering the further development and practical application of this technology.

From a theoretical perspective, ultrasonic welding challenges involve the interdisciplinary integration of materials science, physics, and mechanics. The inherent complexity and uncertainty pose significant challenges to traditional theoretical frameworks, necessitating innovative perspectives and solutions. In practical applications, inconsistent welding quality not only compromises product performance and reliability but also leads to increased production costs and resource wastage.

In recent years, scholars both domestically and internationally have conducted extensive research on ultrasonic welding, accumulating substantial survey data and contributions to the literature. Meanwhile, the introduction of relevant policies and regulations has provided crucial support and guidance for this field. However, despite numerous research outcomes, some fundamental issues in ultrasonic welding processes remain unresolved, such as atomic diffusion mechanisms, interface temperature evolution, and precise quality control. The underlying causes and mechanisms behind these challenges still require further investigation. Therefore, this study aims to thoroughly analyze the causes and impacts of ultrasonic welding problems, propose practical solutions, and provide theoretical support and practical guidance for the sustainable development of this field.

### 1.2. Significance of the Study

This study provides a comprehensive analysis of core challenges in ultrasonic welding technology, systematically tracing its theoretical evolution and practical applications. Through integrated methodologies, including molecular dynamics simulations and experimental investigations, researchers conducted rigorous data analysis to derive forward-looking conclusions with significant practical value. These findings not only validate the theoretical foundations for industrial implementation but also open new research pathways, offering crucial references for future studies. This study identifies the key factors affecting welding quality while proposing targeted solutions that enhance both efficiency and product integrity. These strategies will drive sustainable industry growth through improved welding processes. Furthermore, the research provides actionable insights for industry managers and researchers, playing a vital role in optimizing operational practices and fostering healthy development within the ultrasonic welding sector.

## 2. Model Simulation

The ultrasonic molecular dynamics simulation steps are shown below.

### 2.1. Establishment of Simulation Model

Molecular simulation is a crucial research method that studies molecular structures and properties through computer simulations, including Molecular Mechanics (MM), Monte Carlo (MC) simulations, and molecular dynamics simulations. Molecular Mechanics (MM) is a computational method that describes molecular structures and energies based on classical mechanics, typically used for optimizing molecular geometries and calculating conformational energies. Monte Carlo (MC) simulations utilize random sampling to explore the thermodynamic properties and equilibrium states of systems, making them particularly suitable for studying phase transitions and adsorption processes. In contrast, Molecular Dynamics (MD) simulations solve Newton’s equations of motion to track the time evolution of atomic trajectories, providing insights into dynamic processes and non-equilibrium phenomena.

For multi-atomic molecular systems, if all intermolecular interaction potentials—such as bond stretching potential and bond angle bending potential—are described by potential functions without constraints, the numerical solution of the system’s motion equation is essentially identical to that of single-atom systems. Common MD numerical integration methods include the Verlet algorithm, the predictive correction algorithm, and its derivatives like the frog-jump method and velocity Verlet method. De et al. [1] proposed an implicit integration scheme based on variational integrators for the Discrete Element Method (DEM). This approach not only handles dynamic problems but also degrades into an energy minimization scheme under quasi-static conditions. Following Hamiltonian principles, it derives motion equations by identifying stationary points in the action. The application scope of variational integrators is expanded through Lagrange-D’ Alembert principles, making them applicable to systems with non-conservative forces (e.g., dissipative forces). This is particularly important for particle material simulations where inter-particle contact typically exhibits dissipative characteristics. A time-discrete framework was proposed to lay the foundation for applying the Quasi-Concrete (QC) method to particle systems. This enables full-resolution particle simulations in certain regions while employing more efficient continuum descriptions in others. The proposed integration method is benchmarked against the existing velocity Verlet method, and its equivalence in single collision, long-term stability, and statistics is verified.

Molecular dynamics simulation is based on classical Newtonian mechanics, and the evolution trajectory of the system is tracked by solving the equation of motion of atoms numerically:

$$m_{i}\frac{d^{2}r_{i}}{dt^{2}}=F_{i}=-\nabla V(r_{i})$$

Here, $m_{i},\;r_{i},\;\mathrm{and}\;V$ represent the atomic mass, the position vector, and the potential energy function, respectively. The simulation adheres to the law of conservation of energy, requiring temperature/pressure regulation to achieve the target ensemble.

Early MD simulations were confined within a fixed spatial volume. According to the law of conservation of energy, the total energy of the simulated system remains constant, allowing no fluctuations. From a statistical mechanics perspective, such MD systems are classified as microcanonical or NVE ensembles. However, many practical chemical and biological systems maintain constant temperatures but exhibit variable total energies when in contact with a constant heat source—these represent canonical systems or NVT ensembles. Furthermore, most real-world or engineered systems possess both fixed temperatures and pressures yet demonstrate variable total energy and volume, constituting NPT ensembles.

Common molecular dynamics software includes LAMMPS and GROMACS. LAMMPS, with its robust parallel computing capabilities, is particularly suitable for simulating large-scale systems. Among the commonly used MD software, LAMMPS (Large-scale Atomic/Molecular Massively Parallel Simulator) is highly regarded for its excellent parallel computing efficiency and flexibility, making it particularly suitable for simulating large-scale metallic and alloy systems. GROMACS, on the other hand, is optimized for biomolecular systems and soft matter, offering high performance in simulating proteins, lipids, and polymers, though it can also handle simpler material systems. When dealing with complex ultrasonic welding systems involving multiple materials or numerous atoms, LAMMPS demonstrates efficient computational performance. GROMACS is primarily used for simulating biomolecules and soft matter systems, though it can also handle simple material systems. This study focuses on plastic welding, emphasizing intermolecular interactions, and dynamic processes.

Different metals possess distinct crystal structures, with common examples including Face-Centered Cubic (FCC), Body-Centered Cubic (BCC), and Hexagonal Close-Packed (HCP). Determining a metal’s crystal structure forms the foundation for constructing atomic models. To determine the unit cell dimensions, lattice constants can be obtained through a literature review or experimental measurements.

### 2.2. Core Framework of Molecular Dynamics Simulation

#### 2.2.1. Potential Function Selection

Potential function is the physical basis of molecular dynamics simulation. The accuracy of potential function determines the reliability of simulation results through mathematical formula to quantify the interactions between atoms.

The EAM (Embedded Atomic Method), a widely used potential energy model for simulating material properties like metals and alloys, is based on the concept of electron density. It treats metal atoms as embedded within surrounding electron clouds, where interatomic interactions depend not only on spatial proximity but also on the distribution of electron clouds. This approach allows the EAM potential function to describe the nature of metallic bonds more accurately. Compared with potential functions that only consider two-body interactions, the EAM accounts for multi-body interactions, better aligning with the actual interatomic interactions in metallic systems and effectively reflecting the structural and performance characteristics of metals.

In contrast, Tersoff [2] developed a general form of empirical interatomic potential functions applicable to multi-component systems, primarily designed for covalent materials such as silicon and carbon. Unlike the EAM potential, the Tersoff potential represents a three-body function based on bond order concepts. It describes interactions in covalent bond systems through bond length, bond angle, and the spatial distribution of neighboring atoms. The key advantage of this potential lies in its dynamic reflection of chemical environment changes around atoms, enabling an accurate depiction of bond formation and breaking. By introducing bond angle dependencies, it precisely characterizes the directionality of covalent bonds. To enhance performance in molecular dynamics simulations, conventional models with overly steep cutoff functions often require optimization with smoother parameters. Compared to quantum mechanical methods, the Tersoff potential significantly reduces computational costs, making it ideal for large-scale simulations of covalent systems.

In potential function research, Mendelev et al. [3] utilized first-principles calculations (VASP software) to obtain critical properties of Al-Mg alloys (e.g., lattice parameters, compound formation energy), combined with molecular dynamics simulations to validate solid–liquid phase equilibrium. They proposed a staged fitting strategy: first optimizing the pure metal potential function (based on existing Al/Mg potential functions) to ensure accurate elemental melting points, then incorporating liquid solution energy data and dilute solid solution formation energy through iterative refinement of cross-potential parameters. The study revealed that potential functions fitted solely for solid-state properties could not correctly predict solid/liquid lines in the Al-Mg phase diagram. Therefore, liquid thermodynamic data were incorporated into the fitting process to ensure accuracy of solid–liquid interface properties. This determined that liquid solution energy data must be integrated into alloy potential function development as a prerequisite for simulating solidification phenomena.

Faken et al. [4] developed a common neighborhood analysis method that systematically investigates local atomic structures using 3D computer graphics. By analyzing radial distribution functions through atomic pair decomposition, this approach directly interprets the functional characteristics of these distributions from an atomic perspective and enables the identification of crystal types. The study demonstrated that while most atoms in copper melts were classified as Face-Centered Cubic (FCC) atoms, stacking faults were still observed—a phenomenon traceable to near-critical nuclei formation.

The choice of an appropriate interatomic potential is not merely a technical step but a foundational decision that directly governs the physical fidelity and reliability of the simulation outcomes discussed in subsequent sections. The conclusions drawn from the material-specific case studies (Section 3 and Section 4)—regarding mechanisms such as atomic diffusion, dislocation dynamics, and phase transformation—are strongly dependent on this choice. For example, the activation energy for diffusion or the critical stress for yielding is not an absolute value but one contingent upon the potential’s parameterization. A potential fitted exclusively to the properties of solid phases may fail to accurately model processes involving significant lattice distortion or local melting at the weld interface. Similarly, the ability of a potential to correctly reproduce stacking fault energies will critically affect the simulation of dislocation-mediated plasticity in FCC metals like Cu and Al. Therefore, discrepancies in reported mechanisms or quantitative values across different studies on similar material systems can often be attributed to the use of different empirical potentials. The reliability of any MD study hinges on the demonstrated accuracy of its chosen potential for the specific properties and conditions under investigation.

#### 2.2.2. Boundary Conditions Design

In molecular dynamics simulation, the common types of boundary conditions are Periodic Boundary Conditions (PBC), Non-Periodic Boundary Conditions (NPB), and mixed boundary conditions. Different types of boundary conditions have different principles and application scenarios.

Periodic Boundary Conditions (PBC): These are the most widely used boundary conditions in molecular dynamics simulations. Under PBC, system boundaries are interconnected to form an infinitely repeating three-dimensional grid. This means when a particle exits one boundary, it immediately enters the opposite boundary, creating the illusion of an infinite system. Such boundary conditions effectively prevent boundary effects and are commonly employed to study macroscopic systems, including fluid dynamics and material properties such as crystal structures.

Non-Periodic Boundary Conditions (NPB): These apply to isolated systems such as individual molecules or clusters. In such cases, the system has no boundary connections. Particles that reach the boundary will not re-enter the system, requiring special treatment of particles at the boundary—such as applying fixed forces or implementing reflections.

Mixed boundary conditions: These combine the characteristics of periodic and non-periodic boundary conditions, with parameters tailored to specific simulation requirements. For example, when simulating a two-dimensional film, one might apply periodic boundary conditions in the horizontal plane while implementing non-periodic boundary conditions in the vertical direction.

#### 2.2.3. Optimization of Time Step Length

In molecular dynamics simulations, the selection of time steps plays a crucial role in simulation accuracy. The motion states of molecules change rapidly within extremely short periods, and appropriate time steps can accurately capture these changes, making simulation results closer to real-world scenarios. Excessive time steps may reduce both simulation accuracy and stability. Therefore, it is essential to strike a balance between computational efficiency and precision. For high-precision requirements, smaller time steps are often necessary; when computational resources are limited and strict precision demands are not critical, appropriately increasing time steps is acceptable, provided that simulation stability and result reliability are maintained.

Tersoff [2] developed a general form of empirical interatomic potential functions applicable to multi-component systems. This approach extends traditional single-component potential functions by enabling interpolation for heteronuclear bonds, thereby broadening their application scope in molecular dynamics simulations. In these simulations, the smoothness of potential functions is critical for accurate results. Conventional models often employ overly steep cutoff functions that require optimization with smoother parameters. This improvement significantly enhances the model’s performance in molecular dynamics simulations, particularly when dealing with complex structures or extended simulation durations.

**Figure 1:**
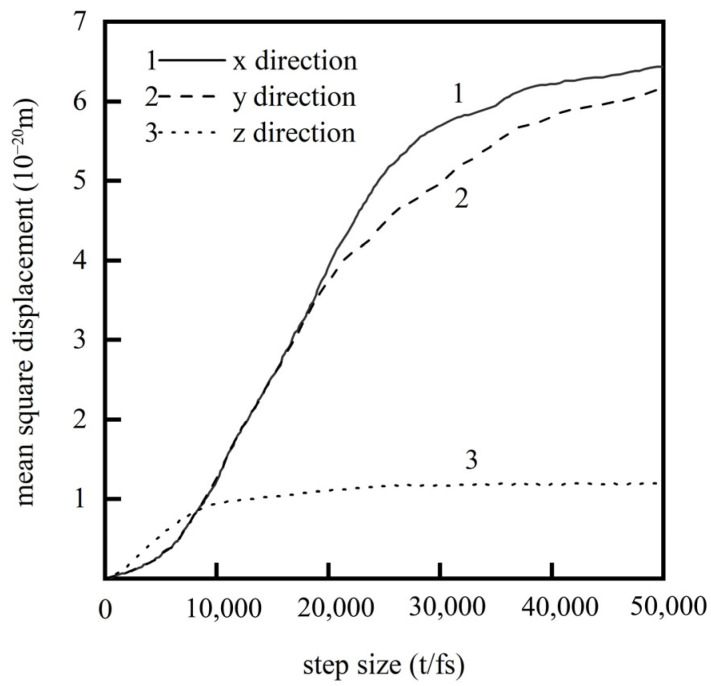
The curve of the mean square displacement of solder atoms varying with the time step [5].

Cheng Hongtao et al. [5] employed the Modified Embedded Atom Method (MEAM) potential energy model to describe intermetallic interactions, elucidating the diffusion behavior of brazing alloys in base materials at atomic scale. Their study conducted detailed analysis of diffusion mechanisms across different orientations: surface diffusion mechanisms dominated in x and y directions with comparable rates, while z-directional bulk diffusion exhibited significantly slower rates, as shown in Figure 1. Simulation results demonstrated that as the reaction reached equilibrium, the Mean Square Displacement (MSD) showed a linear increase with reaction time. After approximately 9000 simulation steps, diffusion rates declined due to the formation of intermetallic compound layers that hindered further diffusion.

### 2.3. Limitations of Molecular Dynamics and Multi-Scale Modeling Approaches

While MD simulations provide unparalleled atomic-scale insights, it is crucial to critically assess their limitations, the most significant of which is the vast spatiotemporal scale gap. Conventional MD operates at the nanosecond (ns) to microsecond (µs) timescale and nanometer (nm) length scale. In stark contrast, experimental ultrasonic welding processes occur over milliseconds to seconds and involve components on the micrometer to millimeter scale. This disparity of several orders of magnitude means that MD cannot simulate a complete, macroscopic welding cycle.

Therefore, the primary value of MD in this context is not to replicate the entire experiment but to act as a “computational microscope” that reveals the fundamental initiation mechanisms, such as the very first atomic displacements, the nucleation of dislocations at the interface, the initial stages of atomic inter-diffusion, and the dynamic evolution of the interface under extreme shear and pressure. These mechanisms are often the root cause of macroscopic weld quality but are exceedingly difficult to observe directly in experiments. To bridge this scale gap, the computational materials science community has developed several strategies, among which multiscale modeling stands as the most comprehensive and necessary framework.

## 3. Principle and Advantage of Ultrasonic Welding Technology

### 3.1. Overview of Ultrasonic Welding

Ultrasonic welding utilizes thermal energy generated by high-frequency vibrations to achieve fusion. As a solid-state welding technique, it operates below the melting point of metals during the process. This method can bond both same-materials and dissimilar materials, particularly excelling in welding soft and highly thermally conductive substances like aluminum, copper, and nickel. Key advantages include rapid processing speed, minimal environmental impact, durable weld points, and user-friendly operation.

### 3.2. Physical Mechanism of Ultrasonic Welding

#### 3.2.1. Three-Stage Coupling of Frictional Heat Generation, Plastic Flow, and Interfacial Diffusion

During the frictional heating phase of ultrasonic welding, ultrasonic vibrations transmitted through the welding head create high-frequency relative motion at the workpiece interface. The microscopic irregularities on the surface—such as tiny protrusions and depressions—interact to generate frictional forces. Generally, higher vibration frequencies and amplitudes enhance frictional resistance, thereby accelerating heat generation. Meanwhile, appropriate pressure ensures sufficient contact area between workpieces, effectively promoting the occurrence of friction-induced heating.

The plastic flow stage begins when the interfacial temperature reaches a critical threshold, triggering material deformation and transitioning into plastic flow. At this phase, atoms or molecules acquire sufficient energy to move and rearrange with relative freedom. Under combined ultrasonic vibration and pressure, the material flows along the interface and stress direction, filling interfacial voids and defects while enhancing both contact area and bonding strength between components.

Interface diffusion, based on plastic flow, involves the gradual diffusion of atoms or molecules at material interfaces over time, thereby enhancing bonding between materials. As a fundamental material transfer phenomenon driven by atomic/molecular thermal motion, ultrasonic welding accelerates this diffusion process through high-temperature conditions and ultrasonic vibrations. This combined action enables the materials on both sides of the interface to progressively fuse into a unified structure.

#### 3.2.2. Quantitative Description of Acoustic Softening Effect and Strain Rate Sensitivity

The acoustic softening effect is a significant phenomenon in ultrasonic welding. During the process, the introduction of ultrasound alters material mechanical properties, specifically reducing yield strength and rheological stress—effectively “softening” the material. This occurs because the high-frequency vibrations of ultrasound influence microstructural features like dislocation movement within materials, promoting dislocation multiplication, motion, and annihilation. Consequently, the material’s ability to resist deformation is diminished.

Strain rate sensitivity refers to a material’s responsiveness to strain rate changes during plastic deformation under external forces. In ultrasonic welding, the dynamic stress and strain generated by high-frequency vibrations make material strain rate sensitivity crucial for weld quality. Specifically, ultrasonic welding utilizes high-frequency vibration waves transmitted to the surfaces of two workpieces. Under pressure, these surfaces rub against each other, creating molecular-level fusion through interfacial melting. The material’s strain rate sensitivity fundamentally determines its plastic deformation behavior under dynamic loads, which directly impacts the strength and integrity of welded joints.

## 4. Factors Affecting Ultrasonic Welding Effect

### 4.1. Grain Boundaries

#### 4.1.1. Cu-Cu

**Figure 2:**
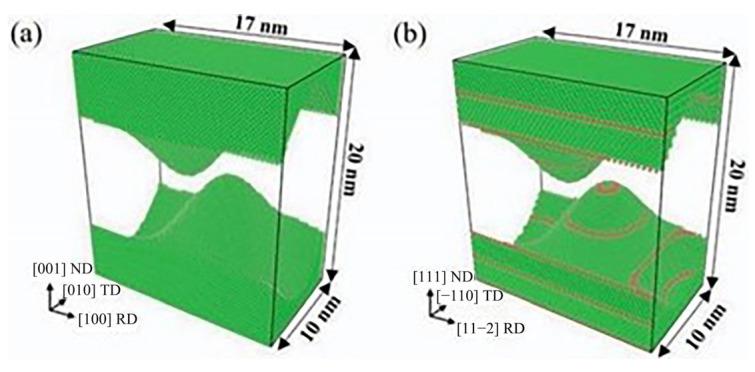
Constructed Cu interatomic layer models with distinct grain orientations and grain boundary structures: (**a**) coarse-grained copper (cg-Cu) and (**b**) nanotwisted copper (nt-Cu) models [6].

Ma et al. [6] investigated the anisotropic deformation mechanisms and mechanical properties of nanotwisted copper (nt-Cu). Using a Cu coating with parallel nanotwisted layers aligned with ultrasonic vibration direction as an intermediate layer, they analyzed how the nt-Cu intermediate layer affects welding quality and deformation mechanisms under different welding pressures. Through Atomsk software, they constructed Cu interatomic layer models with distinct grain orientations and grain boundary structures, including coarse-grained copper (cg-Cu) and nanotwisted copper (nt-Cu) models. This design accurately simulates twin boundary migration behavior in nt-Cu. Figure 2a shows the single-crystal model of cg-Cu without grain boundary structures, used to simulate coarse-grained copper behavior. Figure 2b presents the nt-Cu model where red atomic layers represent twin boundaries (TBs) oriented along the [111] direction perpendicular to the normal direction (ND), which aligns with actual coating structures and authentically reflects the microstructure of nt-Cu.

Experimental and molecular dynamics simulations demonstrate that under lower welding pressures, the non-eutectic copper (nt-Cu) intermetallic layer primarily achieves deformation and twinning reduction through twin boundary migration. This mechanism effectively mitigates work hardening during welding, concentrating deformation at the weld interface to significantly enhance joint strength. At 8 psi welding pressure, the joint strength shows the most substantial improvement of 26.75% compared to conventional coarse-grained copper. As welding pressure increases, the deformation mechanism of non-eutectic copper shifts to transverse and penetration dislocation mechanisms. The interaction between dislocations and twin boundaries forms non-coherent twin boundaries and 9R phases, which reduces the effectiveness of work hardening and strengthening in the interfacial region.

**Figure 3:**
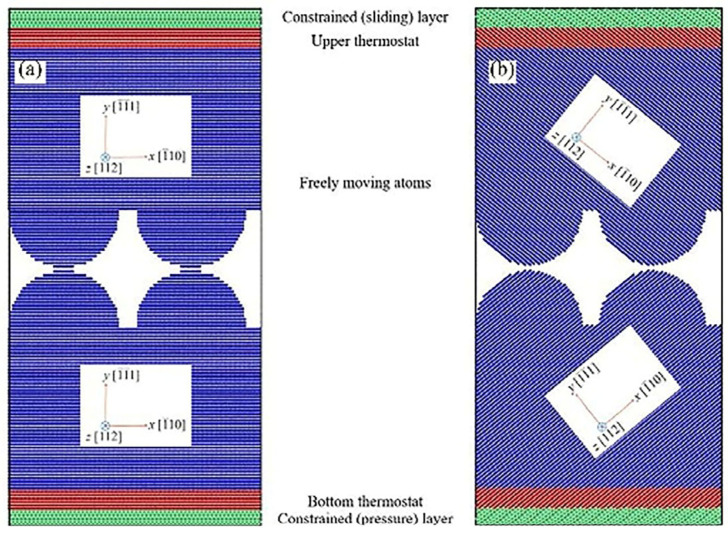
Schematic diagrams of two atomic models: (**a**) the model demonstrates no lattice misalignment and (**b**) the model features a unique 78.46° lattice misalignment [7].

**Figure 4:**
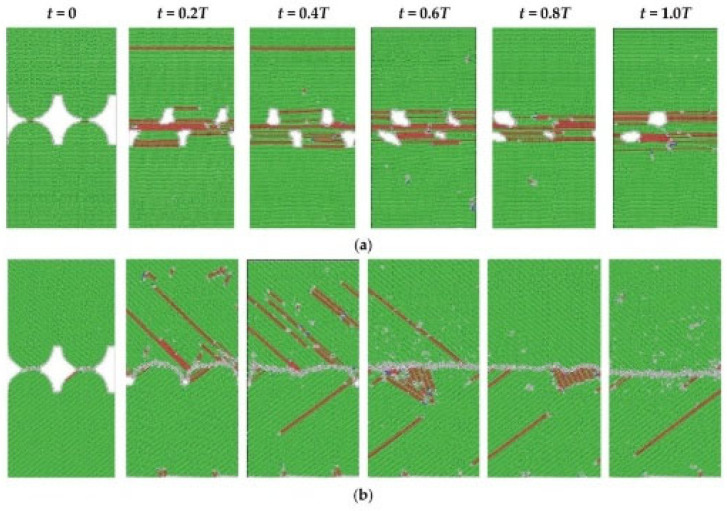
Snapshots of the atomic structures after equilibrium at the same temperature but at different times: (**a**) the model demonstrates no lattice misalignment and (**b**) the model features a unique 78.46° lattice misalignment [7].

Nazarov et al. [7] employed molecular dynamics simulations to investigate the effects of lattice orientation on ultrasonic welding processes using two distinct atomic models, as illustrated in Figure 3. The left panel demonstrates no lattice misalignment, while the right panel features a unique 78.46° lattice misalignment. This configuration facilitates understanding of how different lattice orientations influence phenomena such as interfacial sliding, interface migration, and pore healing. The atomic structures were analyzed using common neighbor analysis (Figure 4), which reveals the evolution of crystal defects during welding. The distribution of atoms with Hexagonal Close-Packed (HCP) coordination vividly illustrates the nucleation and glide of dislocations at the interface. This direct visualization confirms that plastic deformation is primarily accommodated through dislocation activity, which is crucial for achieving atomic-level bonding.

**Figure 5:**
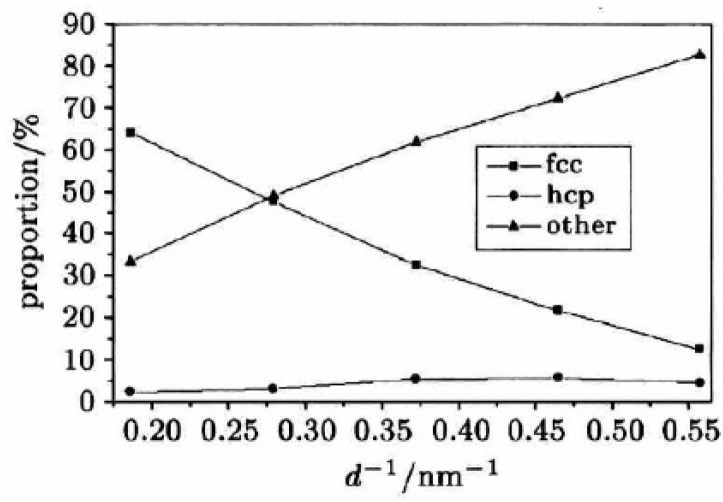
The relationship between the structure of nanocrystalline samples and the grain size [8].

Wen Yuhua [8] constructed nanocrystalline copper samples with varying average grain sizes (ranging from 1.79 nm to 5.38 nm) using molecular dynamics methods, followed by detailed atomic structure analysis. The results showed that as grain size decreased, the proportion of Face-Centered Cubic (FCC) atoms within grains significantly decreased, while the proportion of other-type atoms at grain boundaries increased markedly, as shown in Figure 5. The average energy of atoms within grains rose, whereas that at grain boundaries declined. Notably, the Young’s modulus of nanocrystalline copper was lower than conventional polycrystalline copper and gradually decreased with reduced grain size. The strength and flow stress of nanocrystalline copper also decreased with decreasing grain size, contradicting the strengthening mechanisms observed in traditional coarse-grained materials. A comprehensive analysis revealed that plastic deformation in nanocrystalline copper primarily occurs through grain boundary slip, grain boundary movement, and grain rotation. At low strain (<5%), dislocation motion plays a limited role; however, at higher strain (>5%), dislocation motion becomes dominant, and its influence intensifies with increasing grain size.

Li You [9] systematically elucidated the plastic deformation behavior and mechanisms of bimetallic copper at the nanoscale through molecular dynamics simulations and experimental studies, particularly revealing the influence patterns of different grain boundaries on material mechanical properties. The research also highlighted that molecular dynamics simulations employ computer-aided modeling of atomic nucleus movements to calculate the structure and properties of multi-body systems composed of atomic nuclei and electrons. Each nucleus is treated as a particle moving under the empirical potential fields provided by other nuclei and electrons, governed by Newtonian laws. The core concept involves constructing a particle system to simulate microscopic phenomena, where continuous media are conceptualized as systems composed of N atoms or molecules. Interactions between particles can be derived through methods like quantum mechanical potential function derivatives, while classical Newtonian mechanics establishes motion equations for particles (mathematical models). Numerical solutions of these equations reveal particle trajectories in phase space, from which macroscopic dynamic and static characteristics are determined using statistical physics principles.

#### 4.1.2. Al-Al

Mostafavi et al. [10] developed a single-crystal aluminum model with various crystal orientations (face-centered cubic structure) to simulate the compression, sliding, and cooling phases during ultrasonic welding. The study investigated how crystal orientation, sliding speed, and compression rate affect atomic diffusion at aluminum crystal interfaces. Tensile tests were conducted to evaluate joint load-bearing capacity, establishing correlations between interfacial structures and mechanical properties.

The study revealed that crystal orientation significantly affects interfacial diffusion efficiency and joint strength. Specific orientations (e.g., 100/100) exhibit enhanced diffusion coefficients exceeding 40% due to high lattice matching, resulting in higher tensile strength. Sliding speed demonstrates a critical threshold: insufficiently low speeds lead to inadequate diffusion, while excessively high speeds induce interfacial disorganization. Appropriate compression rates are essential as excessive compression inhibits diffusion layer formation. Frictional heating serves as the driving force for interfacial diffusion, where temperature elevation softens materials and promotes dynamic recrystallization to strengthen joints. By optimizing crystal orientation compatibility with process parameters, the formation of brittle Intermetallic Compounds (IMCs) can be suppressed, thereby improving welding reliability in aluminum stranded wire applications such as new energy vehicle battery connections.

#### 4.1.3. Cu-Al

**Figure 6:**
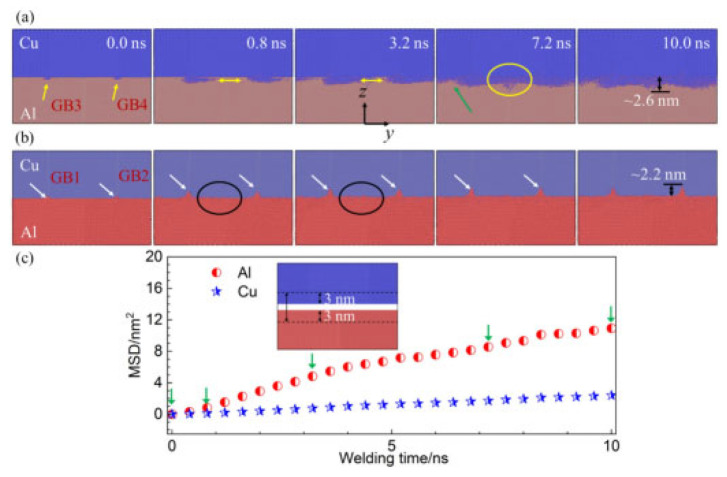
Diffusion of Cu and Al atoms in the bimorph Cu/Al joint: (**a**) Cu atoms diffusing into the Al matrix; (**b**) Al atoms diffusing into the Cu matrix; and (**c**) the average displacement of Al atoms and Cu atoms with increasing welding time [11].

**Figure 7:**
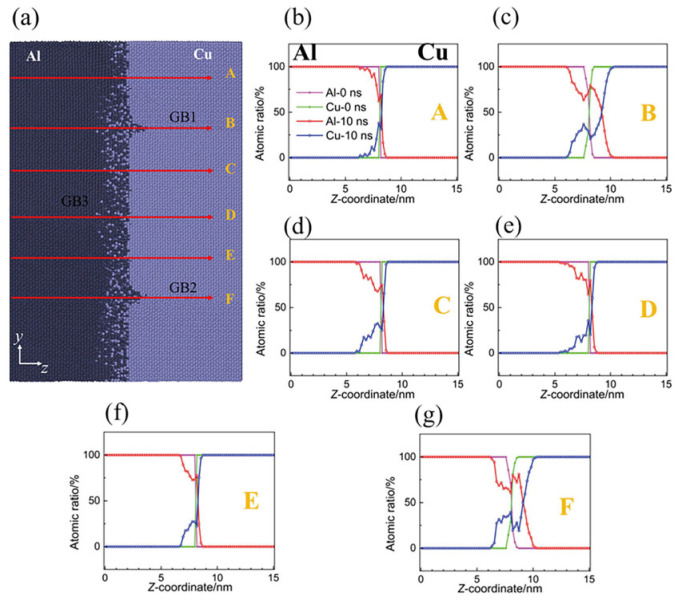
Diffusion results of Cu and Al atoms: (**a**) snapshots of the Cu/Al joint at the end of welding and (**b**–**g**) atomic ratios as functions of Z coordinates of Al and Cu atoms [11].

Yang et al. [11] investigated diffusion processes in Cu/Al duplex joints using molecular dynamics simulations, focusing on typical high-angle Grain Boundary (GB) characteristics. Their study revealed that grain boundaries facilitated the migration of both copper and aluminum atoms, particularly enhancing copper atom penetration into the aluminum matrix. The mean square displacement analysis demonstrated that aluminum atoms exhibited more active movement within the base metal compared to copper atoms, as illustrated in Figure 6. The team analyzed mechanisms including atomic rearrangement, dislocation motion, stacking fault formation, and dynamic recrystallization. During compression and initial welding stages, lattice mismatch and grain distortion caused interface-induced compression of aluminum atoms, leading to dislocation and vacancy formation. In later welding phases, plastic deformation predominantly occurred within the aluminum matrix, manifesting as dislocations, stacking faults, and grain boundary slip. Notably, ultrasonic welding combined shear effects with grain boundary slip to induce interfacial grain recrystallization. Additionally, increasing vibration frequency significantly enhanced diffusion between Cu/Al joints by expanding the number of amorphous regions. The final diffusion results for Cu/Al atoms are presented in Figure 7.

#### 4.1.4. Ni-Ni

Van et al. [12] employed molecular dynamics simulations to investigate low-temperature elastoplastic deformation in Ni nanocrystalline samples with varying average grain sizes (3–5 nm). These polycrystalline specimens, formed through nucleation of different crystal seeds, exhibited random spatial and orientation distributions. The volume modulus and Young’s modulus were calculated from stress–strain curves, with particular attention to the onset of plastic deformation. At higher loading conditions, significant differences in plastic behavior were observed compared to coarse-grained counterparts. The study proposed a mechanism involving grain boundary viscosity to explain deformation mechanisms such as grain boundary sliding and motion, establishing a linear relationship between strain rate and the inverse of grain size.

### 4.2. Temperature

#### 4.2.1. Cu-Al

Samanta et al. [13] developed a hierarchical multiscale approach integrating experimental studies with molecular dynamics simulations. The experimental phase employed ultrasonic welding equipment to bond three layers of aluminum with a copper layer, where STEM and EDS analyses measured interfacial diffusion layer thickness. The molecular dynamics simulation component constructed a nanoscale Al-Cu interface model incorporating atomic potential (EAM) to describe interatomic interactions, combined with classical diffusion theory for computational diffusion coefficient determination. This multiscale methodology integrates atomic-level simulation results from molecular dynamics (e.g., diffusion rates) into macroscopic diffusion models to predict diffusion layer thickness.

By numerically integrating the diffusion coefficient at different sound velocities and considering the influence of sinusoidal variation in sound velocity during ultrasonic vibration, the simulation results show that the solid diffusion coefficient is related to temperature, pressure and transverse sound velocity. The higher the temperature and pressure, the higher the diffusion rate caused by ultrasonic waves, resulting in a larger diffusion layer thickness.

Li et al. [14] investigated the influence of temperature on aluminum–copper interface diffusion bonding using Molecular Dynamics (MD) simulations and Embedded Atom Method (EAM) potential energy. The results demonstrated that during diffusion bonding, Cu atoms predominantly diffuse toward the aluminum side, with the interfacial thickness varying proportionally to temperature—increasing as temperature rises. The interfacial region becomes disordered during this process. Notably, Cu atoms diffuse extensively into the aluminum matrix at low concentrations, while Al atoms exhibit minimal penetration into the copper matrix even at high concentrations. This study identified an optimal temperature range of 750–800 K for aluminum–copper interface diffusion bonding, with activation energies of 0.77 eV and 0.50 eV for aluminum and copper, respectively.

**Figure 8:**
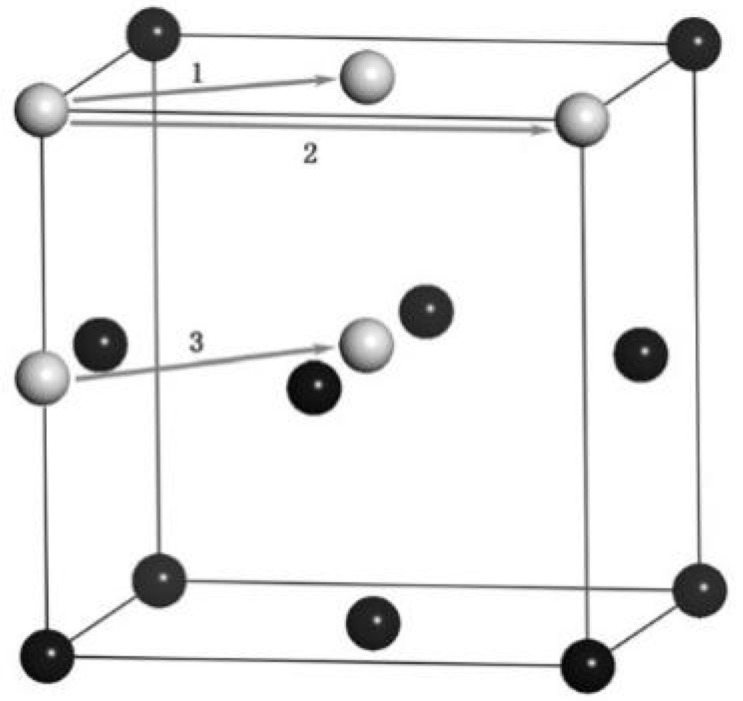
Three diffusion mechanisms of Cu atoms in Al [14].

During this research, three diffusion mechanisms of Cu atoms in aluminum (Al) lattices were identified, as illustrated in Figure 8. The black spheres represent Al atoms, while gray spheres denote Cu atoms. Mechanism 1 corresponds to nearest neighbor hopping, mechanism 2 corresponds to second-nearest neighbor hopping, and mechanism 3 corresponds to interstitial hopping. The first two mechanisms are vacancy diffusion processes. Cu atoms diffuse to the aluminum side, occupy Al lattice positions, then hop to adjacent vacancies. The third mechanism involves Cu atoms diffusing to the aluminum side as octahedral interstitial atoms before moving to another octahedral vacancy. Between 0 and 6 ns, the hopping frequency of Cu atoms in Al was measured, totaling 19 jumps: 15 to nearest neighbors, 3 to second-nearest neighbors, and 1 as an octahedral interstitial atom. Notably, the primary diffusion mechanism is nearest neighbor hopping. The static potential energy barriers for these mechanisms were analyzed using the Non-Equilibrium Binding Energy (NEB) method, which generates a series of intermediate configurations between initial and final states. By minimizing the energy of this sequence, the NEB method identifies the lowest-energy path (MEPs). The calculated energy barrier for interstitial hopping stands at 3.51 eV, exceeding the other two mechanisms. This validation confirms that nearest neighbor hopping is the dominant diffusion mechanism.

**Figure 9:**
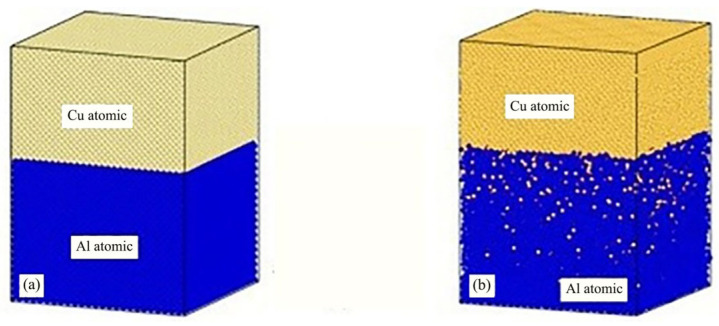
The effects of holding temperature and soaking time on interfacial diffusion: (**a**) initial model and (**b**) Cu/Al diffusion after being held at 800 K for 1.2 ns [15].

Han Xuejie et al. [15] investigated the effects of holding temperature and soaking time on interfacial diffusion and mechanical properties of Cu/Al films through molecular dynamics simulations. The atomic schematic diagram is shown in Figure 9. Results indicated that more copper atoms migrated into the aluminum side than aluminum atoms into the copper side. Copper atoms diffused deeply into the aluminum layer, while aluminum atoms only diffused at the interface. Aluminum’s diffusion coefficient was higher than that of copper. At lower temperatures, the diffusion between copper and aluminum was minimal, but significantly improved at 800 K. Therefore, 800 K was selected as the optimal holding temperature for simulating interfacial diffusion. As soaking time increased, the transition layer thickness grew initially but stabilized afterward. The Cu/Al film achieved optimal mechanical properties when maintained at 800 K for 1.2 ns.

Liu Hao and colleagues [16] employed molecular dynamics simulations to model the diffusion welding processes of copper (Cu) and aluminum (Al). Their study analyzed post-weld specimen evolution during unidirectional tensile testing, using Radial Distribution Functions (RDFs) and bond pair analysis techniques to investigate structural transformations in the intermetallic transition layer under varying cooling rates. The research revealed that higher cooling rates preserved the original disordered structure of the transition layer, while lower cooling rates induced a structural transition from disordered to Face-Centered Cubic (CCD) lattice.

Tensile simulations were conducted on copper–aluminum diffusion-welded specimens and compared with those of single-crystal copper and aluminum specimens of similar dimensions. The results showed that the post-welded specimens exhibited lower strength than their single-crystal counterparts, with stress–strain curve characteristics differing from those of single-crystal materials. This revealed the effects of slip bands and interfacial layer defects on material properties. Bond pair analysis was employed to characterize atomic bonding configurations within the interfacial layer. The method uses four parameters (α, β, γ, δ) to describe the bond states between atoms, further revealing local structural features of the interfacial layer. Additionally, it was found that the cooling rate significantly influences the degree of orderliness in the interfacial layer.

Jin Yuhua and colleagues [17] developed a detailed welding overlap model for friction stir welding, employing a combined approach of shrinking boundary conditions and periodic boundary conditions. Their study not only investigated the effects of holding temperature on atomic diffusion at the Cu-Al interface but also further explored the role of holding time. Through precise control of both temperature and duration, they systematically investigated diffusion behavior under various conditions. The research established 800 K as the optimal holding temperature, where the interfacial transition layer thickness was optimal and mechanical properties were excellent. Using the DXA method, they calculated dislocation lengths and conducted detailed analysis of dislocation evolution during plastic deformation, revealing the dominant role of Shockley-type dislocations. The study also elucidated the formation, propagation, and ultimate fracture of microcracks on the aluminum side during tensile processes, uncovering the microscopic mechanism of interfacial failure. Furthermore, by tracking the movement trajectories of individual copper atoms, they confirmed that the diffusion mechanism in aluminum primarily follows the nearest neighbor hopping principle, providing specific proportional data (82.4%). However, a comparative analysis of the literature reveals discrepancies in the reported dominant diffusion mechanisms and quantitative activation energies for Cu-Al systems. For instance, while Li et al. [14] identified nearest-neighbor hopping as the primary mechanism with activation energies of 0.77 eV (Al) and 0.50 eV (Cu), other studies suggest a more complex interplay of mechanisms or report different energy values.

These differences can be reconciled by considering the variations in simulation methodologies. The choice of interatomic potential is critical, as different parameterizations of the EAM potential can yield varying estimates for migration energy barriers. Furthermore, the interface structure plays a decisive role: simulations involving high-angle grain boundaries [11] provide preferential diffusion channels that can lower the effective activation energy compared to simulations of more ideal single-crystal interfaces [14]. The temperature range used for extracting activation energies can also influence the result, as different mechanisms may be thermally activated at different rates. Finally, the statistical significance of the observed events, which is affected by simulation duration and system size, can bias the reported dominant mechanism. Therefore, the apparent discrepancies in the literature likely reflect the sensitivity of atomic diffusion to specific simulation conditions and local atomic environments, rather than fundamental contradictions. This underscores the importance of carefully matching the simulation setup (e.g., potential choice, interface model) to the specific physical scenario one aims to model.

#### 4.2.2. SWNTs-Ni

**Figure 10:**
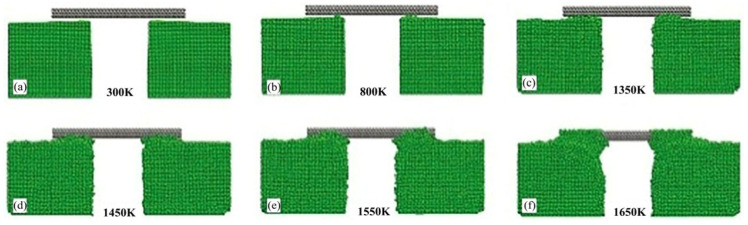
Snapshots of the welding system at different temperatures: (**a**) 300 K; (**b**) 800 K; (**c**) 1350 K; (**d**) 1450 K; (**e**) 1550 K; (**f**) 1650 K [18].

**Figure 11:**
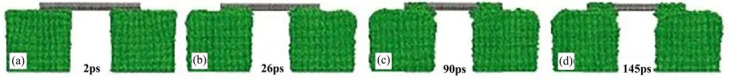
The interfacial microstructure of the welding system at different times at 1500 K: (**a**) 2 ps; (**b**) 26 ps; (**c**) 90 ps; (**d**) 145 ps [18].

This study on SWCNTs-Ni, while involving a carbon nanostructure, provides valuable insights into the ultrasonic welding of metal matrices in composite systems, particularly demonstrating the “acoustic softening effect” below the melting point of the nickel phase. Liu et al. [18] investigated the composite mechanism between Single-Walled Carbon Nanotubes (SWNTs) and Ni matrix using Molecular Dynamics (MD) and experimental methods. They established a horizontally aligned composite system composed of a metal electrode and SWNT subsystem for simulation studies, which comprehensively described the kinetic processes at atomic scale. The simulations demonstrated that bimetallic welding could be achieved below Ni’s melting point (1726 K). At 800 K, only a small number of Ni atoms slowly migrated onto the SWNTs. As temperature increased to 1650 K, more Ni atoms departed from equilibrium positions and underwent intense vibrations, ultimately completely enveloping the SWNTs, as shown in Figure 10. The study also examined the influence of welding duration on the interface, with Figure 11 showing similar phenomena to Figure 10. The complete welding process took approximately 145 ps.

This phenomenon shows that nano-welding can be completed at a temperature below the melting point of Ni, proving the existence of the “acoustic softening effect”.

#### 4.2.3. TC4-TC4

**Figure 12:**
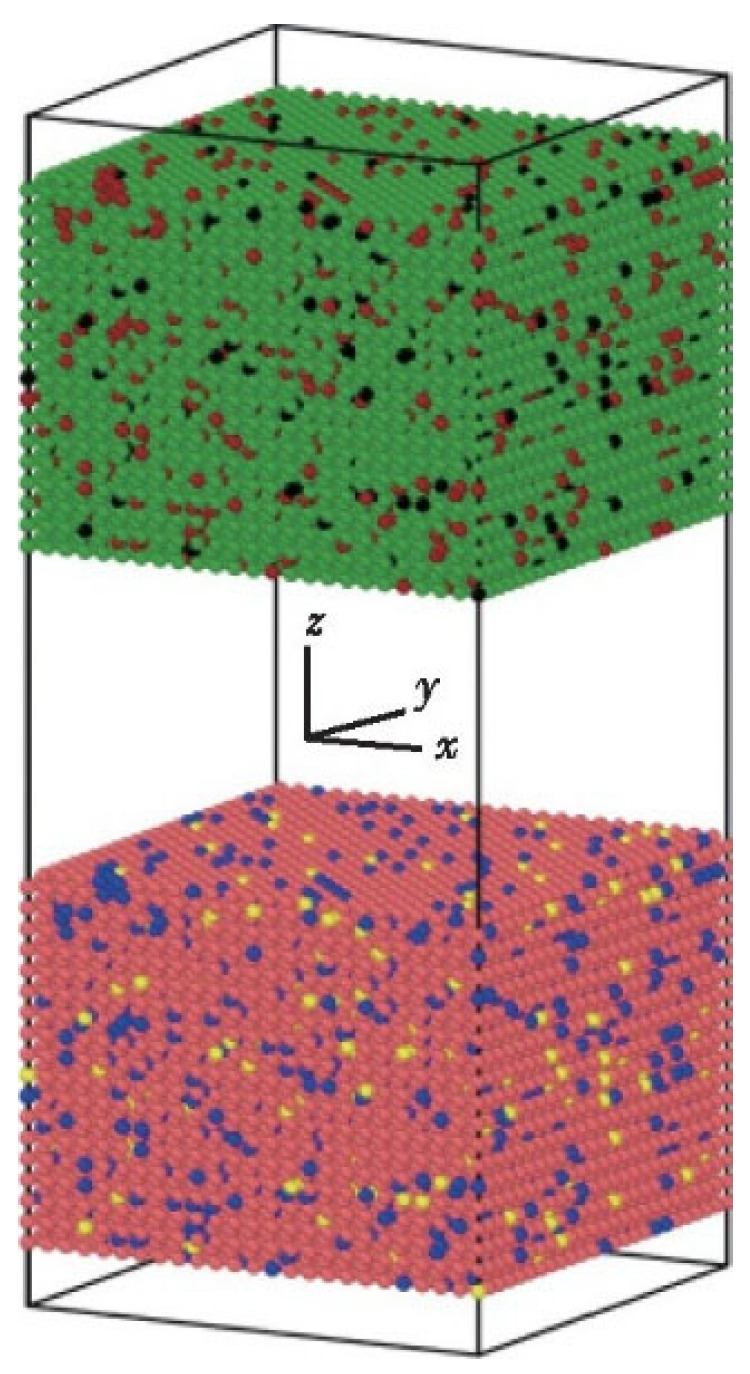
The initial 3D model of the diffusion bonding process of the TC4 titanium alloy [19].

Zhang Shun et al. [19] conducted a molecular dynamics simulation study on the diffusion bonding process of TC4 titanium alloy (Ti-6Al-4V), with the model architecture shown in Figure 12. The green and pink atoms represent titanium, while blue and dark red atoms denote aluminum, with black and yellow atoms representing vanadium. A hybrid potential function combining Embedded Atomic Potential (EAM) and Morse potential was employed to describe interatomic interactions. Radial distribution function analysis revealed atomic concentration patterns during diffusion, demonstrating lattice structure evolution mechanisms. Through controlled parameter experiments, systematic investigations were performed on how temperature, pressure, holding time, and cooling rate affect diffusion bonding width. The optimal process parameters were determined as follows: a critical holding temperature of approximately 1100 K at 800 ps holding time under constant pressure, with vanadium atoms showing intermediate diffusion capability between titanium and aluminum. Simulation results indicated faster titanium atom diffusion near the interfaces, slower aluminum atom diffusion, and vanadium atoms exhibiting intermediate diffusion rates during TC4 titanium alloy diffusion bonding.

### 4.3. Surface Morphology

#### 4.3.1. Ti-Al

**Figure 13:**
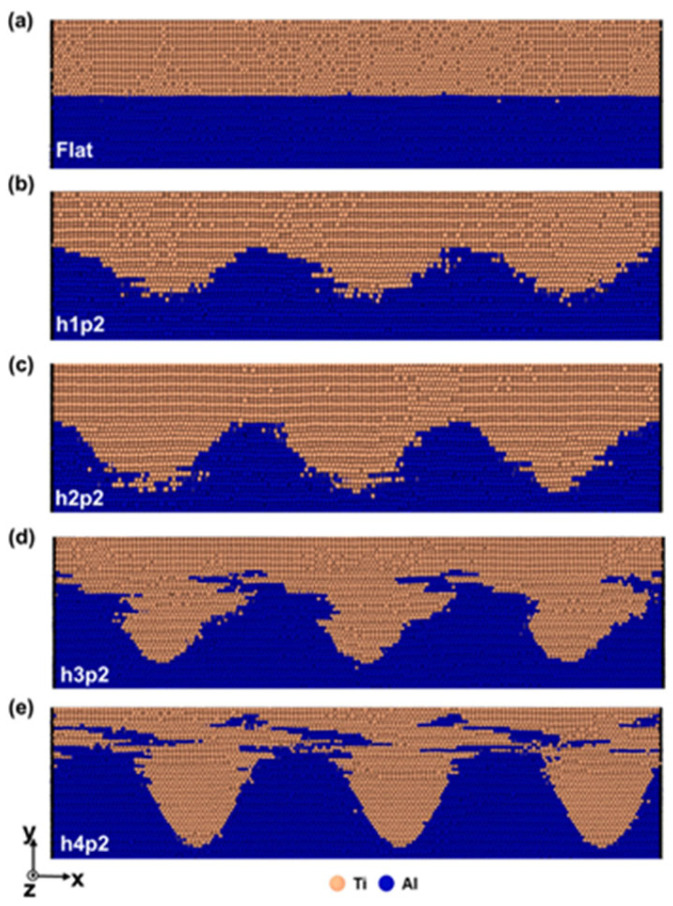
The atomic structures changed with the h-plane and four sinusoidal interface models in the same period: (**a**) flat; (**b**) h1p2; (**c**) h2p2; (**d**) h3p2; (**e**) h4p2 [20].

**Figure 14:**
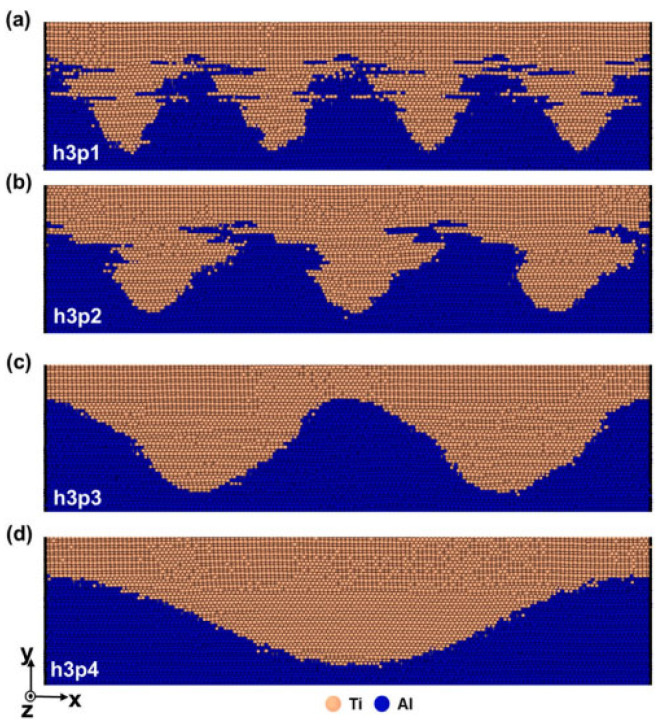
A sinusoidal interface model with the same height but changing the spatial period: (**a**) h3p1; (**b**) h3p2; (**c**) h3p3; (**d**) h3p4 [20].

Moon et al. [20] used molecular dynamics simulation to study the mechanical mixing and deformation behavior of ultrasonic welded Ti/Al, analyzing the influence of interface shape on interface mixing. To investigate the impact of interface morphology, models with both flat and sinusoidal interfaces were constructed (Figure 13 and Figure 14). The simulation results demonstrate that the sinusoidal geometry actively promotes mechanical mixing at the interface. As the curve height increases, the expanded interfacial area disrupts periodicity and facilitates a more chaotic intermixing of Ti and Al atoms, which is a key driver for forming a sound weld.

Simulation results indicate that, as the height of the sinusoidal curve increases, mechanical mixing at the interface becomes more active. The expanded interface area reduces the spatial periodicity of mechanical mixing at the interface, leading to a phase transition occurring at the sinusoidal curve interface during welding. Under vibration load, dislocation mismatch formed at the interface undergoes slip along the Shockley split slip planes, transforming HCP Ti into FCC Ti. Experimental findings reveal that, although sinusoidal Ti/Al interfaces exhibit higher welding strength than planar interfaces, this strength is independent of mechanical mixing degree. Instead, it is influenced by interactions between the dislocation walls near the interface during UW processes, stacking fault tetrahedrons, and lattice dislocations.

#### 4.3.2. Cu-Cu

Ma et al. [6] developed welding surfaces with varying microroughness scales, systematically analyzing the combined effects of deformation heat and the influence of shear strain and strain rate on recrystallization and weld quality. By constructing hemispheres of different diameters (representing microroughness levels from microscale to macroscale), they conducted simulations to investigate how material flow and interfacial shear deformation are affected during welding processes.

**Figure 15:**
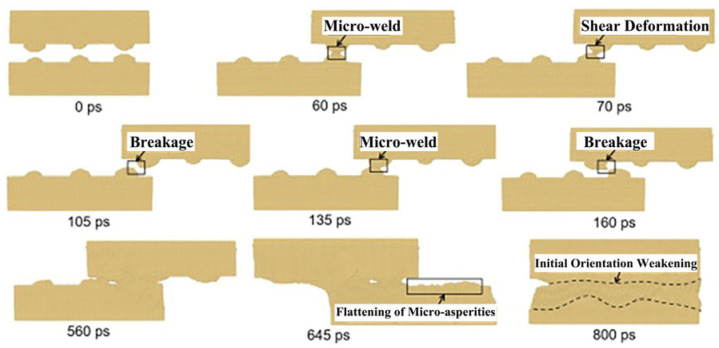
The welding formation of Cu-Cu joints under micro-roughness joints [6].

**Figure 16:**
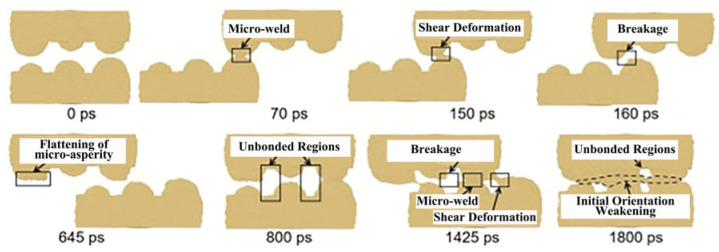
The welding formation of Cu-Cu joints under large and micro roughness joints [6].

Molecular dynamics simulations demonstrate that weld formation is a dynamic process involving micro-connections, tearing, fracture, flattening, and weld propagation, as illustrated in Figure 15 and Figure 16. The reduction in micro-surface roughness accelerates the flattening process and intensifies shear deformation at tight contact zones. According to computational models showing roughness-induced recrystallization, smaller micro-protrusions during shear deformation facilitate coupling between high shear strain and low strain rate. This achieves a maximum shear strain of 1.57 and a minimum average strain rate of 346/s, thereby reducing the critical recrystallization strain and expanding the dynamic recrystallization region in ultrasonic welding.

#### 4.3.3. Cu-Al

Song Cheng [21] constructed a molecular dynamics model of copper–aluminum heterojunctions to elucidate the dynamic contact behavior of interfacial protrusions during ultrasonic welding initiation. Simulation results revealed that these protrusions undergo a cyclic process of “contact–tear–break–reconnect” under ultrasonic vibration. This dynamic evolution mechanism overcomes the limitations of traditional experimental methods in capturing transient interfacial behaviors, providing an atomic-scale theoretical basis for understanding micro-zone bonding formation in early welding stages. Innovatively combining experimental observations with molecular dynamics simulations, the study uncovered the core mechanism by which copper nanoparticles reduce welding energy. Simulations demonstrated that nanoparticles optimize interfaces through two pathways: first, acting as “fluid mediators” to fill surface micro-protrusion gaps and accelerate interfacial flattening; and second, enhancing atomic diffusion driving forces to promote metallurgical bonding. This multi-scale research approach quantitatively explains the physical essence of nanoparticle-assisted energy reduction from microscopic perspectives like atomic migration and dislocation movement for the first time. By calculating evolution curves of interfacial potential energy, kinetic energy, and frictional heat, the study quantified the conversion paths of mechanical energy, thermal energy, and interfacial bonding energy during welding for the first time. The findings reveal that nanoparticles can achieve more uniform distribution of interfacial frictional heat and reduce energy losses caused by localized hot spots, offering theoretical guidance for optimizing low-energy welding processes. This research breaks through the limitation of traditional molecular dynamics simulations confined to static interface analysis, establishing a new analytical framework for energy regulation in dynamic welding processes.

#### 4.3.4. Mg-Al

Feng Mengnan [22] established a multiscale correlation between three-dimensional molecular dynamics models and macroscopic finite element simulations, enabling cross-scale analysis from atomic diffusion behavior to macroscopic thermal evolution. By constructing a Mg-Al interface friction model, he overcame the limitations of traditional single-scale simulations. The study systematically revealed the synergistic mechanisms of process parameters on interfacial atoms: temperature increases (300–500 K) tripled the diffusion coefficient of Mg atoms; non-steady-state turbulent diffusion occurred when relative friction velocity reached 60 m/s; and vacancy concentration increased to 3% enhanced Al vacancy mobility by 47%. He innovatively proposed a theoretical model explaining how surface roughness affects diffusion pathways, demonstrating that nano-scale protrusions reduce effective contact area by 35% while boosting local diffusion flux by 80%. The quantitative relationship between interfacial vacancy concentration gradients and diffusion activation energy was established, providing theoretical foundations for suppressing brittle compound growth.

**Figure 17:**
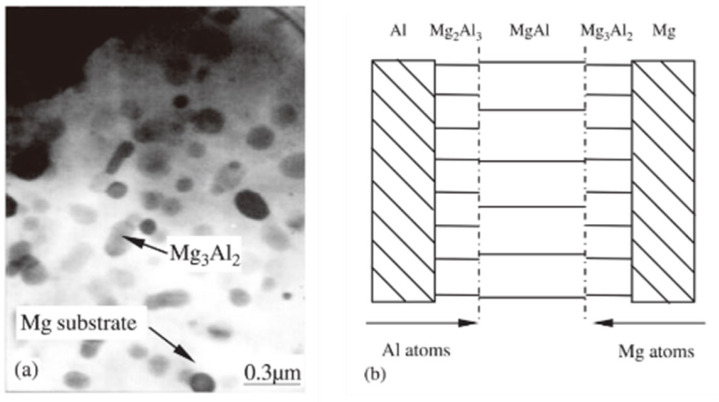
The interfacial structure of magnesium–aluminum diffusion: (**a**) TEM morphology of the Mg_3_Al_2_ phase near the transition region on the Mg side; (**b**) interfacial diffusion zone model [23].

Peng et al. [23] investigated the interfacial structure of magnesium–aluminum diffusion welded joints using Scanning Electron Microscopy (SEM), Electron Probe Microanalysis (EPMA), and Transmission Electron Microscopy (TEM). The results revealed that the interface region of the magnesium–aluminum diffusion welded joint consists of three distinct zones: a magnesium transition zone, an intermediate diffusion zone, and an aluminum transition zone. The atomic concentration distribution within these diffusion zones exhibited three distinct phases: Mg_2_Al_3_, Mg_3_Al_2_, and MgAl, as illustrated in Figure 17b. This configuration clearly defines the locations of Mg_2_Al_3_, Mg_3_Al_2_, and MgAl phases within the diffusion zone. Notably, TEM observations confirmed that the Mg_3_Al_2_ phase is predominantly distributed in the magnesium-side transition zone, as shown in Figure 17a.

#### 4.3.5. Fe-W

**Figure 18:**
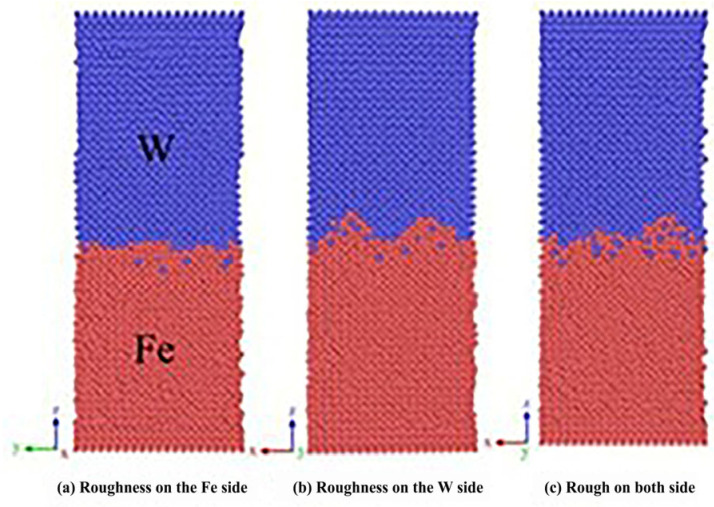
Three simulated interface roughness conditions at 6 ns: (**a**) Fe-side rough; (**b**) W-side rough; (**c**) bilateral rough [24].

Song Kuijing et al. [24] established an interface structure model for Fe(100)/W(100) low-index crystal planes. Simulation results revealed distinct asymmetric diffusion phenomena at the Fe/W interface, primarily characterized by W atoms diffusing into the Fe matrix, while Fe atoms struggle to penetrate the W bulk, with this disparity becoming more pronounced over time. The diffusion activation energies for Fe and W atoms at the Fe/W interface were calculated as 1.326 eV and 0.842 eV, respectively. It was observed that both temperature and pressure enhance diffusion layer thickness, with temperature demonstrating superior enhancement effects compared to pressure. Within the 1123–1323 K range, diffusion layer thickness increased significantly with rising temperature, particularly reaching 1.1 A between 1223 K and 1323 K. Simulations of three distinct interface roughness conditions (Fe-side rough, W-side rough, and bilateral rough) revealed that W-side roughness notably increased diffusion layer thickness. All three rough interfaces were filled with Fe atoms, indicating Fe’s greater susceptibility to deformation than W. While increased interface roughness promotes diffusion layer growth within certain ranges, excessive roughness may compromise interfacial bonding, as illustrated in Figure 18.

### 4.4. Time

#### 4.4.1. Cu-Al

**Figure 19:**
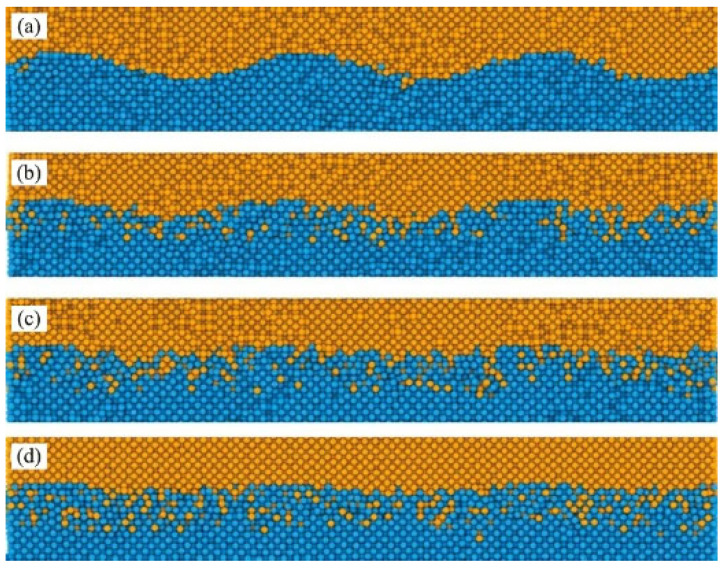
Atomic configurations of the Al/Cu interface at different time: (**a**) 0.1 ns; (**b**) 1 ns; (**c**) 3 ns; and (**d**) 5 ns [25].

Yang et al. [25] investigated atomic diffusion behavior during Al-Cu ultrasonic welding through molecular dynamics simulatios. At 0.1 ns (Figure 19a), the interface began to blur with localized atomic diffusion. As time progressed (Figure 19c,d), Cu diffused significantly more into Al compared to Al diffusing into Cu. This asymmetric diffusion pattern was confirmed by distinct interface changes, aligning with experimental results. The study demonstrated that asymmetric diffusion occurred at the Al/Cu interface during welding, with diffusion layer thickness increasing over time. Additionally, they observed recovery of disordered Al bulk structures under low-temperature conditions.

**Figure 20:**
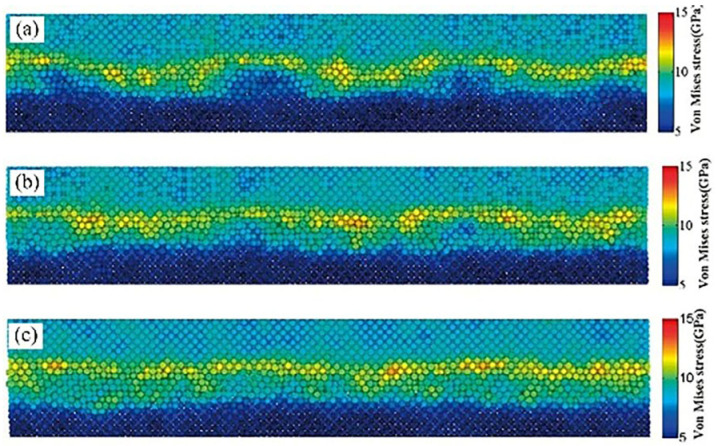
Atomic configurations of the Al/Cu interface at different time: (**a**) 1 ns; (**b**) 3 ns; (**c**) 5 ns [25].

As welding time increases, the thickness of the diffusion layer gradually grows. Notably, during extended welding durations (1 ns), a distinct phase transition phenomenon emerges within the diffusion layer. Despite the relatively low interfacial temperature, the diffusion coefficient remains significantly higher than in steady-state diffusion processes (below 375 K), primarily attributed to shear plastic deformation at the interface. This demonstrates that the diffusion process differs from conventional steady-state diffusion, as illustrated in Figure 20 below.

**Figure 21:**
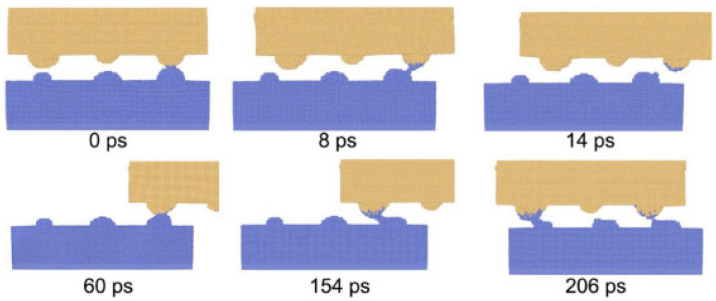
The formation and fracture of micro-welds in the first movement cycle [26].

**Figure 22:**
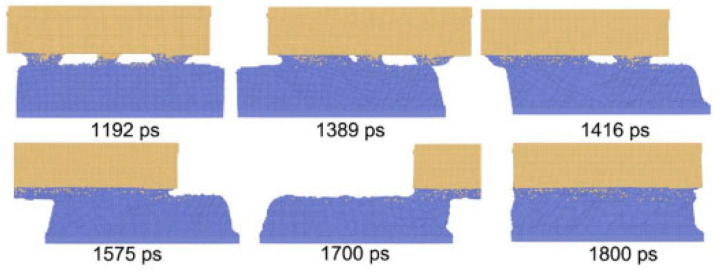
Flatness and weld formation [26].

Ma et al. [26] fabricated a T2 copper–aluminum alloy (Cu-Al) interface using ultrasonic welding equipment, employing Scanning Electron Microscopy (SEM), Backscattered Electron Diffraction (BSEDD), and Transmission Electron Microscopy (TEM) to analyze interfacial morphology, grain orientation, and deformation mechanisms. They developed a Cu-Al surface micro-convex model with hemispherical rough peaks of varying sizes through LAMMPS simulations, which captured atomic-scale dynamics during ultrasonic vibration—including dislocation evolution and micro-weld bead formation/rupture (Figure 21 and Figure 22). The study revealed that micro-weld beads initially formed at high roughness peaks, with plastic deformation concentration on the aluminum side attributed to asymmetric deformation caused by hardness differences between copper and aluminum. The research further investigated how welding duration influenced interfacial bonding morphology, demonstrating the transition from mechanical interlocking to full metallurgical bonding. Quantitative analysis showed a positive correlation between shear strength and welding duration. Microstructural evolution was attributed to dynamic recrystallization under high strain rates, resulting in significantly refined grains on the aluminum side forming a vortex-like structure, while copper grains retained their original morphology. A nanocrystalline-amorphous mixed transition layer at the interface inhibited brittle metal propagation.

#### 4.4.2. SWCNTs-Ni

**Figure 23:**
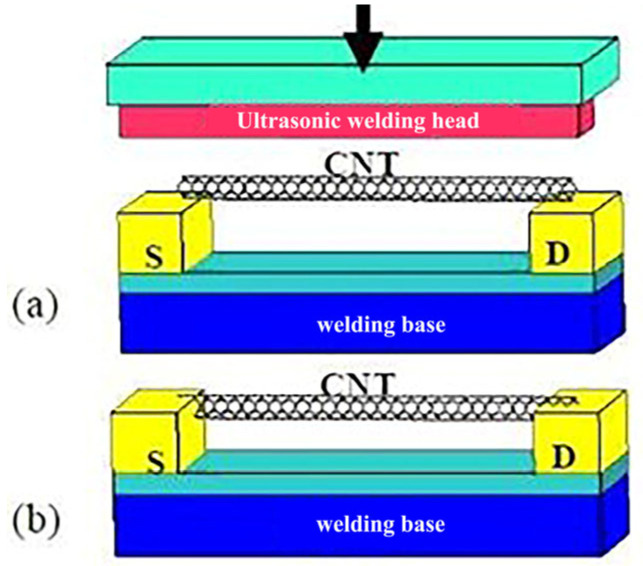
Schematic diagram of ultrasonic nano-welding process: (**a**) before welding; (**b**) after welding [27].

Liu et al. [27] employed molecular dynamics simulations to investigate ultrasonic nanosoldering between Single-Walled Carbon Nanotubes (SWCNTs) and metal electrodes, as illustrated in Figure 23. The nanofuel model system consists of a metal electrode subsystem and a CNT subsystem. A single-crystal aluminum substrate was selected for the metal electrode subsystem, with 1500 atoms arranged in a 6 × 6 × 10 unit face-centered cubic lattice. Simulation results demonstrate that high-frequency ultrasound energy softens the metal and induces plastic deformation under clamping stress, enabling successful ultrasonic welding.

**Figure 24:**
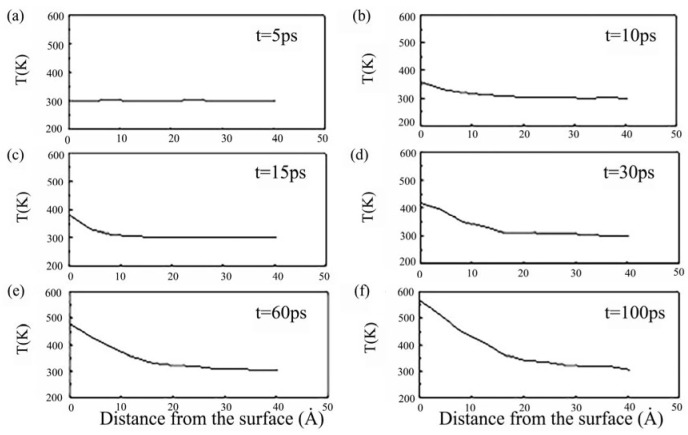
The depth distribution of electrode atomic temperatures with different duration of ultrasonic vibrations: (**a**) 5 ps; (**b**) 10 ps; (**c**) 15 ps; (**d**) 30 ps; (**e**) 60 ps; (**f**) 100 ps [27].

As shown in Figure 24, the surface exhibits the highest temperature while the bottom reaches the lowest. This indicates that friction energy is first absorbed by surface atoms before being transferred through thermal conduction to deeper atomic layers. The maximum temperature remains below the metal’s melting point, confirming that no melting occurs during welding—a key feature of ultrasonic nanoscale welding technology. Additionally, extending the duration of ultrasonic vibrations enhances the energy intensity of the superimposed ultrasound, enabling more effective softening of electrode atoms.

### 4.5. Displacement Rate

#### 4.5.1. Al-Al

Mostafavi et al. [28] employed molecular dynamics simulations to investigate the mechanical and thermal behavior of the weld joint interface during ultrasonic metal welding. Given that ultrasonic welding involves a force–heat coupling process, the study utilized molecular dynamics methods to analyze the nanomechanical mechanisms of this complex coupling process at the picosecond time scale. The research also conducted atomic-scale simulations of the microstructure at and around the welding interface.

**Figure 25:**
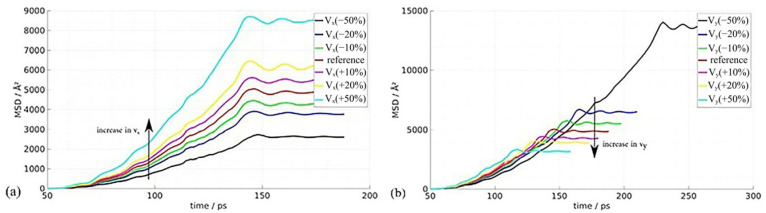
The MSD changes in interface atoms with different process parameters: (**a**) sliding speed; (**b**) the compression ratio [28].

This study investigated the evolution of interfacial temperature during ultrasonic metal welding and analyzed how process parameters influence both interfacial temperature and atomic diffusion behavior. As shown in Figure 25, the interfacial temperature increases significantly with rising sliding speed (vx). The compression ratio (vy) shows minimal effect on interfacial temperature, indicating that sliding speed is the key parameter controlling interfacial temperature (Figure 25a). The figure bottom reveals the time-dependent variation in the Mean Square Root Displacement (MSD) of interfacial atoms. Increased sliding speed enhances atomic diffusion, as evidenced by steeper MSD curves. Conversely, the increased compression ratio (vy) suppresses atomic transfer while having limited impact on the diffusion processes. These findings were compared with the macroscopic experimental results to validate the conclusions.

#### 4.5.2. Ag-Cu

**Figure 26:**
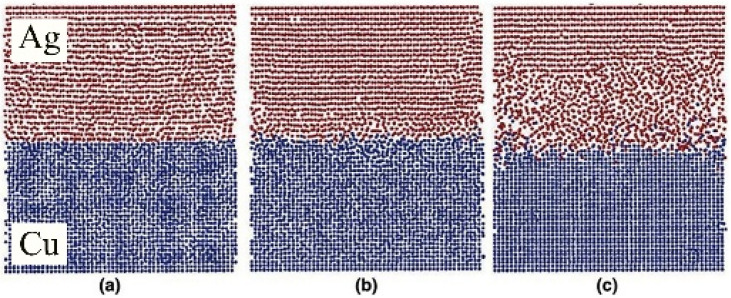
Cross-sectional snapshots obtained under different stresses after 200 fs (blue represents copper atoms; red represents silver atoms): (**a**) 50 Mpa; (**b**) 100 Mpa; (**c**) 150 MPa [29].

**Figure 27:**
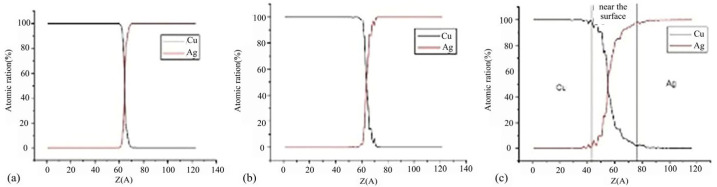
The concentration distributions along the Z direction under different stresses were obtained: (**a**) 50 Mpa; (**b**) 100 Mpa; and (**c**) 150 MPa [29].

Chen et al. [29] investigated the diffusion bonding process and characteristics of the Cu-Ag interface at the atomic level using molecular dynamics methods. They constructed cross-sectional structures obtained under different stress conditions after 200 ps. At 50 MPa stress, no observable diffusion between Cu and Ag was observed, maintaining their Face-Centered Cubic (FCC) structure, as shown in Figure 26a. As illustrated in Figure 26b, only minimal Cu diffusion into Ag occurred at 100 MPa stress. When stress increased to 150 MPa, significant interfacial diffusion emerged, with substantial Cu diffusing into the silver substrate to form a silver-rich interface, as depicted in Figure 26c. The region where both Cu and Ag concentrations exceed 5 Å is defined as the interfacial region. Concentration curves estimate the thickness of this region, as shown in Figure 27. From left to right, the interfacial thickness values are 5 Å, 9 Å, and 27 Å. Figure 27c further reveals that the interfacial region consists of Cu-rich and Ag-rich phases, with the Ag-rich phase thickness exceeding that of the Cu-rich phase. This indicates predominant diffusion from Cu to the Ag substrate.

#### 4.5.3. Cu-Al

**Figure 28:**
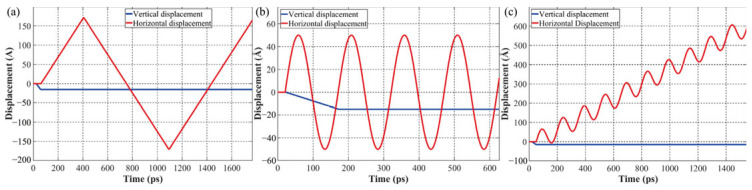
The displacement applied to the wire section: (**a**) DP1; (**b**) DP2; (**c**) DP3 [30].

Long et al. [30] investigated micro-welding point evolution under varying surface topographies and displacement modes through molecular dynamics simulations. Their study revealed that micro-welding points can form or fracture instantaneously. The relative motion between local segments and substrates causes significant deformation or even fracture. The simulations not only captured formation mechanisms but also thoroughly analyzed deformation and failure processes under different configurations. This dynamic behavior analysis provides a comprehensive perspective for understanding micro-welding point dynamics. Three displacement modes (DP1, DP2, DP3) were introduced to simulate real bonding scenarios: DP1 specifically analyzed vertical and horizontal motions affecting formation, deformation, and failure; DP2 examined vibration amplitude effects in regions with negligible sliding displacement; and DP3 simulated simultaneous sliding and vibration at contact edges to study surface morphology changes from repeated micro-welding formation and failure, as illustrated in Figure 28.

Through quantitative analysis of shear stress and equivalent bonding area, this study investigated the effects of material properties, surface morphology, proximity distance, and vibration amplitude on micro-welding point variations. The results demonstrate that these ccc. Analysis of variation scales revealed that small-scale simulations accurately represent macroscopic outcomes, which align with commonly used surface roughness ranges. This comprehensive understanding of micro-welding point variations facilitates the development of optimized control strategies and enhances bonding process performance. An equivalent area-based method for estimating micro-welding point dimensions is proposed, which indirectly reflects changes in joint area and surface morphology while providing a quantitative analytical approach.

### 4.6. Summary of Simulation Conditions and Parameters

To facilitate a direct comparison of the molecular dynamics studies reviewed in this section, Table 1 summarizes the key simulation conditions and parameters employed in the cited literature.

### 4.7. Comparative Analysis of Key Findings

Building upon the descriptive review, Table 2 synthesizes the key findings and atomic-scale mechanisms revealed by these MD simulations, allowing for a clear cross-comparison.

The comparative analysis in the tables above reveals several overarching insights. First, the dominant deformation mechanism is highly scale-dependent: grain boundary-mediated processes (sliding, migration) prevail in nanocrystalline systems (e.g., Ni-Ni [12], fine-grained Cu [8]), while dislocation activity and twin boundary dynamics dominate in larger-grained or single-crystal systems (e.g., Cu-Cu [6,7]). Second, for dissimilar metal joints (e.g., Cu-Al), asymmetric atomic diffusion is a universal finding, driven by differences in atomic radius, melting point, and lattice stability. Third, the efficacy of interface structures is clear: engineered geometries like nanotwins in Cu [6] or sinusoidal interfaces in Ti-Al [20] can channel deformation and recrystallization in beneficial ways, thereby improving weld quality. Finally, while temperature is a universally critical parameter, MD simulations uniquely highlight the coupled role of mechanical driving forces (strain, stress, acoustic softening) in enabling solid-state bonding at temperatures far below the melting point. This synthesis underscores the power of MD in moving beyond phenomenological description to reveal the fundamental, multi-physics mechanisms that control ultrasonic welding.

## 5. Results and Future Prospects

This comprehensive review has synthesized the molecular dynamics (MD) simulation research in the field of metal ultrasonic welding. Through a detailed examination of the literature, we have delved into the core theoretical issues, demonstrating that MD simulations provide an indispensable atomic-scale perspective on the complex physical mechanisms governing the process. Key insights revealed through this review include the critical roles of atomic diffusion, dislocation dynamics, grain boundary interactions, and the acoustic softening effect in determining weld formation and quality. Furthermore, the review has systematically analyzed how MD simulations elucidate the influence of crucial factors such as grain boundaries, temperature, surface morphology, time, and displacement rate on the welding outcome.

The integration of findings from studies on various material systems (e.g., Cu-Cu, Al-Al, Cu-Al) underscores the power of MD in not only visualizing and explaining experimental phenomena but also in predicting optimal process windows and guiding parameter optimization. This aligns with the stated objective of providing robust theoretical support for practical applications, offering a pathway to reduce experimental iterations and R&D costs.

A particularly powerful application of multiscale modeling is the coupling of MD with the Phase-Field (PF) method and Finite Element Models (FEMs). MD can provide the crucial kinetic parameters—such as interface mobility, grain boundary energy, and diffusion coefficients—required by PF models to simulate the dynamic microstructural evolution (e.g., recrystallization and grain growth) in the thermomechanically affected zone of the weld. Simultaneously, MD-derived data on interface strength and constitutive behavior can be integrated into FEM simulations. This enables the prediction of macroscopic weld attributes, such as joint morphology, residual stress distribution, and ultimate shear strength, based on fundamental atomic-scale processes, thereby creating a truly predictive digital twin of the welding process.

Looking forward, a paramount challenge for MD simulations is to move beyond idealized clean interfaces and incorporate the complex chemistry of real-world surfaces. Realistic interface effects such as hydrogen embrittlement, native oxide layers, and organic contaminants or impurities are often decisive for weld quality but remain largely unaddressed in current atomistic studies. Future research must pioneer simulations that include these factors. For instance, simulations could investigate how hydrogen atoms segregate to grain boundaries and dislocation cores under ultrasonic stress, potentially leading to embrittlement. Similarly, modeling the fragmentation and dissolution of thin oxide films on metals like aluminum and copper is crucial to understand the conditions for achieving metallurgical bonding. Furthermore, the presence of surface hydrocarbons or other impurities could significantly alter frictional behavior and atomic diffusion. Developing robust simulation methodologies to account for these phenomena is essential for bridging the gap between idealized models and industrial practice, ultimately enabling the prediction and mitigation of welding defects.

This comprehensive review has synthesized the research status of Molecular Dynamics (MD) simulation in the field of metallic ultrasonic welding. The body of work demonstrates MD’s unparalleled capability in elucidating atomistic mechanisms such as atomic diffusion, dislocation dynamics, grain boundary interactions, and the acoustic softening effect. However, to advance the field from mechanistic understanding to predictive science, several concrete scientific challenges must be addressed, pointing to key future research directions.

(1) Development and Validation of Machine-Learning Interatomic Potentials (ML-IAPs): The reliability of MD simulations is fundamentally constrained by the accuracy of empirical potentials. The development of ML-IAPs trained on high-quality quantum mechanical data represents a paradigm shift. Future work should focus on creating and validating ML-IAPs for complex, multi-component alloy systems relevant to welding. These potentials promise to achieve near-density functional theory (DFT) accuracy while maintaining computational efficiency, enabling realistic simulations of chemical complexity, phase transformations, and defect interactions at the weld interface that are currently beyond the reach of traditional potentials.

(2) Multi-Scale Frameworks to Bridge Time and Length Scales: As highlighted in Section 2.3, the inherent scale gap of MD remains a primary challenge. Future efforts must prioritize the development and application of multi-scale modeling frameworks. This includes using coarse-grained MD to simulate larger material volumes, employing accelerated dynamics (e.g., hyperdynamics, metadynamics) to access experimental timescales for rare events like nucleation, and, most critically, establishing robust MD-to-continuum coupling schemes. The integration of atomistically informed parameters—such as interface strength, diffusion coefficients, and constitutive laws—into Crystal Plasticity Finite Element Models (CPFEM) or phase-field models will be essential for predicting the macroscopic performance and reliability of welded joints.

(3) Strategies for Experimental Validation and Integration: The ultimate test of any simulation lies in its agreement with experimental reality. A critical future direction is the design of dedicated experiments for the direct validation of MD predictions. This can be achieved through synergies with in situ characterization techniques, such as High-Resolution Transmission Electron Microscopy (HR-TEM) for observing interface structures, or synchrotron X-ray diffraction for measuring stress and strain fields at the micro-scale. Furthermore, MD should be used not in isolation but as an integral part of an iterative loop with continuum models and experiments, where simulation insights guide experimental design, and experimental data, in turn, validates and refines the simulation models.

## Figures and Tables

**Table 1:** Summary of Molecular Dynamics Simulation Conditions for Ultrasonic Welding Studies.

Material System	Reference	Potential Function	Ensemble	Temperature (K)	Time/Length Scale	Key Parameters Varied
Cu-Cu	[6]	Not Specified	NVT	300	Nanoscale	Welding pressure, grain structure (cg-Cu vs. nt-Cu)
Cu-Cu	[7]	EAM	NVT	300	ns/nm	Lattice misorientation (0° vs. 78.46°)
Cu-Cu	[8]	Not Specified	NPT	300	ns/nm	Grain size (1.79–5.38 nm)
Al-Al	[10]	Not Specified	NVT	300–600	ns/nm	Crystal orientation, sliding speed, compression rate
Cu-Al	[11]	EAM	NVT	300	ns/nm	Grain boundary presence, vibration frequency
Cu-Al	[14]	EAM	NPT	300–800	ns/nm	Temperature
Cu-Al	[15]	Not Specified	NPT	400–800	ns/nm	Holding temperature, soaking time
Ni-Ni	[12]	Not Specified	NPT	Low Temp	ns/nm	Grain size (3–5 nm)
SWNTs-Ni	[18]	Not Specified	NVT	300–1650	ps/nm	Temperature, welding time
Ti-Al	[20]	Not Specified	NVT	300	ns/nm	Interface morphology (flat vs. sinusoidal)
Mg-Al	[22]	Not Specified	NVT	300–500	ns/nm	Temperature, friction velocity, vacancy concentration
Fe-W	[24]	Not Specified	NPT	1123–1323	ns/nm	Temperature, pressure, interface roughness

**Table 2:** Summary of Key Findings and Mechanisms from MD Simulations of Ultrasonic Welding.

Material System	Reference	Key Findings and Atomic-Scale Mechanisms
Cu-Cu	[6]	nt-Cu interlayer enhances strength via twin boundary migration at low pressure but shifts to dislocation-based mechanisms at high pressure.
	[7]	Lattice misorientation promotes interface migration and pore healing via dislocation activity.
	[8]	Strength decreases with grain size reduction; deformation shifts from grain boundary slip/rotation (small grains) to dislocation motion (larger grains).
Al-Al	[10]	Crystal orientation is critical; high lattice matching (e.g., 100/100) yields >40% higher diffusion and strength. A critical sliding speed exists for optimal diffusion.
Cu-Al	[11,14,25]	Asymmetric diffusion is universal: Cu diffuses more actively into Al. Grain boundaries and higher temperatures significantly enhance diffusion. Dominant mechanism is nearest-neighbor hopping.
	[21]	Surface protrusions undergo “contact–tear–break–reconnect” cycles. Nanoparticles act as “fluid mediators” to enhance bonding.
Ni-Ni	[12]	At the nanoscale (3–5 nm grains), deformation is governed by grain boundary sliding and motion, not dislocations.
SWNTs-Ni	[18]	Welding occurs below Ni melting point (acoustic softening). Ni atoms envelop SWNTs at high temperatures (~1650 K).
Ti-Al	[20]	Sinusoidal interfaces promote mechanical mixing and phase transition (HCP Ti to FCC Ti), but strength is governed by dislocation interactions, not mixing degree.
Mg-Al	[22]	Diffusion is enhanced by higher temperature, friction velocity, and vacancy concentration. Surface roughness reduces contact area but boosts local diffusion flux.
Fe-W	[24]	Highly asymmetric diffusion: W diffuses into Fe but not vice versa. W-side interface roughness most effectively increases diffusion layer thickness.

## Data Availability

No new data were created or analyzed in this study.
